# Towards a Cancer Mission in Horizon Europe

**DOI:** 10.1002/1878-0261.12585

**Published:** 2019-10-31

**Authors:** Anton Berns, Ulrik Ringborg, Alexander Eggermont, Michael Baumann, Fabien Calvo, Angelika Eggert, Carolina Espina, Douglas Hanahan, Denis Lacombe, Francesco de Lorenzo, Simon Oberst, Thierry Philip, Joachim Schüz, Josep Tabernero, Julio E. Celis

**Affiliations:** ^1^ The Netherlands Cancer Institute Amsterdam The Netherlands; ^2^ European Academy of Cancer Sciences; ^3^ Cancer Center Karolinska Karolinska University Hospital Stockholm Sweden; ^4^ Gustave Roussy Cancer Campus Grand Paris Villejuif France; ^5^ German Cancer Research Center (DKFZ) Heidelberg Germany; ^6^ German Cancer Consortium (DKTK) Heidelberg Germany; ^7^ Charite‐Universitatsmedizin Berlin Germany; ^8^ International Agency for Research on Cancer (IARC/WHO) Lyon France; ^9^ Swiss Institute for Experimental Cancer Research (ISREC) Federal Institute of Technology in Lausanne (EPFL), and Swiss Cancer Center Leman (SCCL) Lausanne Switzerland; ^10^ EORTC Headquarters Brussels Belgium; ^11^ European Cancer Patient Coalition Brussels Belgium; ^12^ Cancer Research UK Cambridge Centre UK; ^13^ Organisation of European Cancer Institutes (OECI); ^14^ Institut Curie Paris France; ^15^ Medical Oncology Department Vall d’Hebron University Hospital and Institute of Oncology (VHIO) Universitat Autonoma de Barcelona Spain; ^16^ Danish Cancer Society Research Centre Copenhagen Denmark

Ever since former Commissioner for Research Philippe Busquin established the European Cancer Research Area (ECRA) in 2002 (http://europa.eu/rapid/press-release_SPEECH-02-408_en.htm) to address the fragmentation of cancer research in Europe, the cancer community and policymakers have been trying to develop strategies to bridge the gaps between basic/preclinical and clinical research and research and healthcare (Celis and Pavalkis, 2017). These efforts culminated in 2014 with the creation of Cancer Core Europe (Eggermont *et al.*, 2019), a patient‐centred legal structure on therapeutics that currently consists of seven large cancer centres (mainly Comprehensive Cancer Centres (CCCs, institutions that link research with the healthcare system; Saghatchian *et al.*, 2008) across Europe. Inspired by the Cancer Core Europe initiative, a consortium of 10 cancer prevention centres was recently established – Cancer Prevention Europe – to reinforce the complete cancer prevention research continuum (Wild *et al.*, 2019). At present, Cancer Core Europe and Cancer Prevention Europe are in the process of integrating their strategies to create a coherent plan for prevention, early detection and treatment, and efforts are underway to engage the outcomes research geometry and to network with other infrastructures, CCCs, and research and clinical centres across Europe (https://febs.onlinelibrary.wiley.com/toc/18780261/2019/13/3).

To date, several prominent scientific cancer organisations and cancer centres are collaborating to develop a unified insight towards a mission‐oriented approach to cancer. These include Cancer Core Europe, Cancer Prevention Europe, the Organisation of European Cancer Institutes (OECI), the European Organisation for Research and Treatment of Cancer (EORTC), the European Association for Cancer Research (EACR), the EuroTech Universities Alliance, the European Cancer Patient Coalition (ECPC), the European Academy of Cancer Sciences (EACS), and the European Society for Medical Oncology (ESMO); other organisations are expected to join shortly. Getting where we stand today required (a) building communities, (b) working in partnership, (c) engaging key stakeholders, (d) identifying champions among researchers, clinicians, policymakers and patients, (e) organizing science, and (f) providing future perspectives and evidence‐based advice to inform policy (Celis and Heitor, 2019; Celis and Pavalkis, 2017).

The EACS, as an independent organisation composed of eminent oncologists and cancer researchers, has actively supported the creation of a Cancer Mission (Adami *et al.*, 2018) and, as a result, has been requested to coordinate the process of developing such a joint strategy to speak with a single voice. At a recent meeting organised by the EACS in Brussels, most of the organisations mentioned above met to prepare a short document addressing the goals, structuring activities and areas of priority required to accomplish the mission. The text, which was recently sent to the Cancer Mission Board, stated:

‘To have impact on society at large, the Cancer Mission aims at uniting countries to substantially reduce the massive EU cancer burden and improve the quality of life of patients by promoting cost‐effective, evidence‐based best practices in cancer prevention, treatment, and care. Our goal is to achieve 10‐year cancer survival for 3/4 patients by the year 2030. Because cancer mortality provides a more timely assessment of progress which also captures advances in primary prevention, it will be important to document the expected declining trends in age‐standardized mortality in each EU country.

Achieving the goals will require:

- Integrated, networked, and distributed infrastructures needed to reach the critical mass of resources, multidisciplinary expertise, technologies, data, patients and coordinated collaborative projects that are essential to promote science‐driven and social innovations in the era of personalized (precision) cancer medicine (Fröhlich *et al.*, 2018). The latter calls for institutional collaborations as exemplified by Cancer Core Europe (therapeutics), Cancer Prevention Europe or SIOP Europe. By generating a coherent cancer research continuum, the infrastructures will offer innovative approaches for cancer research, links to the healthcare and prevention systems, development of quality‐assured multidisciplinary cancer care, as well as the assessment of long‐term outcomes of prevention and therapeutics. Moreover, they will provide an ecosystem to support education and training, mobility, capacity building and rapid dissemination of information and best practices across the European Union.
- Effective collaboration among all the stakeholders (basic, translational, and clinical researchers, clinical oncologists/healthcare professionals, pathologists, radiation oncologists, surgeons, prevention researchers, epidemiologists, patient organisations, universities, industry and small and medium‐sized enterprises (SMEs), regulatory bodies and funders).
- Coordinated networks of CCCs1Comprehensiveness is designated to those centres that have a well‐established combination of fundamental and translational cancer research, with a sufficiently large portfolio of clinical trials, and multidisciplinary cancer care services covering the care pathway, and linked with primary care. and research and clinical centres across Europe.
- A portfolio of projects/activities across the cancer research/care continuum from basic/preclinical to early clinical, late clinical, and outcomes research leading to recommendations for best practices to treat patients.
- The use of cohesion funds to ensure the successful participation of central and eastern European member states.
- Active involvement of the cancer research, prevention/health care and cancer patient communities at all stages of policymaking.
- Coordinated communication to disseminate information to cancer patient organisations, national cancer societies, the cancer research community, policymakers and society at large.
- Incentives and funding for structuring activities as well as for areas of research priority (see below).

Structuring activities will entail:

- *Creating a partnership model of CCCs and CCCs of Excellence (CCCoE): A network of networks.* There should be at least one CCC in each EU country. Newly‐created and accredited CCCs generated through supportive partnership arrangements should result in manageable networks (ideally serving a population of 5−10 million) of CCC/CCCoE and other centres for innovation and high‐quality multidisciplinary cancer research, cancer care, rehabilitation and prevention. CCCs have an outreach role in sharing best practices with local hospitals and healthcare providers. CCCoE, on the other hand, should serve as sites for advanced infrastructural provisions. OECI and EACS are to be charged with accreditation of the CCC and CCCoE, respectively.
- *Attracting and incentivising the next generation of cancer specialists*. Education, training and early career development for cancer scientists, engineers and physicians (PhD studentships, postdoctoral fellowships, fellowships providing protected time for physician scientists, bridge grants for early career development (comparable to the successful NIH KO8 programs), as well as support for PI projects following the ERC/Synergy concept to generate new insights by “basic and preclinical cancer research” and serve as “feeder” for the programs listed. Also, there is a role for professional organisations (e.g. ECCO, ESMO, ESTRO, ESSO, SIOPE etc.), the EACR and the EACS.
- *Fostering networks of excellence for proof of concept trials and for clinical trials changing practice in prevention, therapeutics and outcomes research*. This will require innovative proof of concept trials embedded in a high‐quality translational research environment to discover the treatments of tomorrow. Attention should be given to rare cancers, comorbidities and specific clinical needs of particular patient groups (e.g. geriatric patients). Innovations generated by early prevention/clinical research must demonstrate added value before use by the healthcare/prevention organisations. For the final assessment of cost‐effectiveness, outcomes research is required.
- *Establishment of a policy board to identify best strategies to implement activities effectively*. Essential tasks include the analysis of funding mechanisms, organizing the science, addressing ethical issues, and the establishment of robust governance structures to achieve the set goals.

Areas of research priority:

- *Fundamental and preclinical research: the engine that fuels innovation in prevention and therapeutics*. Fostering innovative research to identify new causes of cancer, to develop diagnostic markers for detecting premalignant disease, to elucidate the underlying mechanisms of drug and immunotherapy resistance and to identify therapeutic strategies to overcome these.
- *Primary cancer prevention: a pan‐European programme (exposure and lifestyle focused)*. Cancer prevention and implementation research to be concentrated in networks with a wide geographical distribution. Identification of causes of cancer and methodologies to identify individuals at higher risk, combined with initiatives to reduce carcinogenic exposures and encourage a healthy lifestyle while offering equal access to active primary prevention (e.g. vaccination, medical prevention).
- *Early detection for prevention and treatment*. Equal access to effective early diagnosis and treatment programs. Development and dissemination of innovative, more sensitive, and minimally invasive low‐cost methodologies for diagnosing cancers, including premalignant tumours to improve prevention screening and early treatment of cancer.
- *Development of new therapies*. Increase in the number of academia‐initiated clinical trials (including diagnostics, medical oncology, radiation therapy, surgery and multimodal treatment) with a clear aim to improve survival and quality of life, with special emphasis on precision medicine and gender‐specific aspects.
- *Outcomes research including health‐related quality of life aspects*. Outcomes research to assess the effectiveness (decreased mortality, survival benefits, health‐related quality of life), with links to health economics of primary/secondary prevention and therapeutic interventions. Addressing the psychosocial and socioeconomic aspects of cancer, rehabilitation, supportive and palliative care, long‐term follow‐up and survivorship including social consequences (societal acceptance, insurance, employment) and health economics aspects.
- *Big‐data generation and computational science*. Establishment of EU‐wide, shared databases providing access to standardised patient‐derived data and genomic/molecular marker information from wearables. Development of AI paradigms for mining the data to identify new correlations (improvements in predicting response, relapse, and side effects).
- *Fostering preclinical and clinical research in paediatric cancer*. Support the further development of a comprehensive pediatric oncology program by the pediatric oncology community represented by SIOP Europe’.

The document will be updated regularly as new organisations and infrastructures join the platform, and further information becomes available regarding governance and the portfolio of projects and activities.

## Conflict of interest

Carolina Espina and Joachim Schüz state: where authors are identified as personnel of the International Agency for Research on Cancer / World Health Organization, the authors alone are responsible for the views expressed in this article, and they do not necessarily represent the decisions, policy or views of the International Agency for Research on Cancer / World Health Organization.

Michael Baumann states: In the past 5 years, Dr. Baumann attended an advisory board meeting of MERCK KGaA (Darmstadt), for which the University of Dresden received a travel grant. He further received funding for his research projects and for educational grants to the University of Dresden by Teutopharma GmbH (2011–2015), IBA (2016), Bayer AG (2016–2018), Merck KGaA (2014–2030), Medipan GmbH (2014–2018). For the German Cancer Research Center (DKFZ, Heidelberg) Dr. Baumann is on the supervisory boards of HI‐STEM gGmbH (Heidelberg). Dr. Baumann, as former chair of OncoRay (Dresden) and present CEO and Scientific Chair of the German Cancer Research Center (DKFZ, Heidelberg), was or is responsible for collaborations with a multitude of companies and institutions, worldwide. In this capacity, he has signed/signs contracts for his institute(s) and for the staff for research funding and/or collaborations with industry and academia, worldwide. In this role, he was/is further responsible for commercial technology transfer activities of his institute(s), including the DKFZ‐PSMA617 related patent portfolio and similar IP portfolios. Dr. Baumann confirms that none of the above funding sources were involved in the preparation of this paper. Other authors declare no conflict of interest.
